# Incidence and Predictors of Multimorbidity in the Elderly: A Population-Based Longitudinal Study

**DOI:** 10.1371/journal.pone.0103120

**Published:** 2014-07-24

**Authors:** René Melis, Alessandra Marengoni, Sara Angleman, Laura Fratiglioni

**Affiliations:** 1 Department of Geriatric Medicine/Nijmegen Alzheimer Centre 925, Radboud University Nijmegen Medical Centre, Nijmegen, The Netherlands; 2 Geriatric Unit, Department of Clinical and Experimental Science, University of Brescia, Brescia, Italy; 3 Aging Research Center (ARC), Karolinska Institutet (Neurobiology, Care Science and Society Department) and Stockholm University, Stockholm, Sweden; 4 Stockholm Gerontology Research Center, Stockholm, Sweden; INRCA, Italy

## Abstract

**Background:**

We aimed to calculate 3-year incidence of multimorbidity, defined as the development of two or more chronic diseases in a population of older people free from multimorbidity at baseline. Secondly, we aimed to identify predictors of incident multimorbidity amongst life-style related indicators, medical conditions and biomarkers.

**Methods:**

Data were gathered from 418 participants in the first follow up of the Kungsholmen Project (Stockholm, Sweden, 1991–1993, 78+ years old) who were not affected by multimorbidity (149 had none disease and 269 one disease), including a social interview, a neuropsychological battery and a medical examination.

**Results:**

After 3 years, 33.6% of participants who were without disease and 66.4% of those with one disease at baseline, developed multimorbidity: the incidence rate was 12.6 per 100 person-years (95% CI: 9.2–16.7) and 32.9 per 100 person-years (95% CI: 28.1–38.3), respectively. After adjustments, worse cognitive function (OR, 95% CI, for 1 point lower Mini-Mental State Examination: 1.22, 1.00–1.48) was associated with increased risk of multimorbidity among subjects with no disease at baseline. Higher age was the only predictor of multimorbidity in persons with one disease at baseline.

**Conclusions:**

Multimorbidity has a high incidence at old age. Mental health-related symptoms are likely predictors of multimorbidity, suggesting a strong impact of mental disorders on the health of older people.

## Introduction

During the last two decades, research on multimorbidity, defined as the coexistence of a number of chronic diseases in the same individual, has rapidly increased. As chronic conditions are strongly related to aging, the majority of studies focused on the elderly population. However, since now, only selected aspects of multimorbidity were investigated, such as prevalence and consequences of co-existing diseases [1]. These previous reports have clearly shown that multimorbidity affects a large proportion of older persons ranging from 55 to 98% across studies depending on definition, age of the population and data source [2], [3]. It has been established that there is a clear association between increasing number of chronic diseases and disability [4]–[6], poor quality of life [6], [7] and high health care utilization [8], [9].

Development of multimorbidity and identification of possible predictors of multimorbidity has been explored in few studies, which showed that 1-year incidence of multimorbidity (defined as 2 or more new diseases) was 1.3% in the whole population including all ages [10]. A few possible risk factors for multimorbidity were identified, such as increasing age [10] and a low socioeconomic status [10]–[12], whereas a large social network seemed to play a protective role [13]. Recently, the mediating role of smoking and body mass index (BMI) on the educational effect was detected in a German population [11].

In the current study, we aimed to estimate the incidence of multimorbidity, and to identify possible predictors for multimorbidity. Several factors were evaluated, including life-style related factors, medical conditions and biomarkers. As the effect of these factors may depend on the health status of a person, we stratified participants at baseline according to presence or absence of one chronic disease.

## Methods

### Study design and participants

This study gathered data from the Kungsholmen Project (KP), which is a cohort study on aging and dementia carried out in Stockholm, Sweden, in a population of 75 years and older subjects (n = 2368) [14]. The KP was approved by the ethics committee of the Karolinska Institute. Each participant signed a written informed consent. The current study used as sample the 418 participants in the first KP follow up (1991–1993) who were living independently and were not affected by multimorbidity. Among them, 149 did not have any chronic disease and 269 were affected by only one chronic disease. After 3-year follow-up, 28 participants refused to be re-assessed; thus complete data on 390 persons were available for analysis (140 without any chronic disease and 250 with one disease).

### Data collection

At all examinations in the KP the data were collected following the same standardized protocols, including a social interview, a neuropsychological battery and a medical examination [14]. Blood samples were obtained for several laboratory tests. The elderly and their next-of-kin were interviewed by trained nurses using a structured questionnaire on living conditions and social status.

### Chronic diseases

A disease was classified as chronic if it satisfied one of the following criteria: 1. being permanent, 2. caused by non-reversible pathological alteration, 3. requiring rehabilitation, or 4. requiring a long period of care. Chronic conditions were assessed using multiple sources [2]: the diagnoses made by the physicians at each assessment in the KP assessments using data from medical history and medication use, clinical examination, and laboratory testing, and the computerized Stockholm Inpatient Registry. The inpatient registry encompasses all hospitals in the Stockholm area since 1969, and records up to six diagnoses at discharge. To increase completeness of the data on the presence or absence of each disease, in addition to this information we used the ATC [18] coded medication data to establish the presence of cancer, depression, diabetes, hypertension, Parkinson’s disease, and thyroid disease from the use of medication only prescribed for these specific conditions. Also, we used the laboratory data on hemoglobin to establish the presence of anemia using an algorithm classifying males with Hb of <13 g/l and females with Hb of <12 g/l as anemic according to WHO criteria [19]. Dementia diagnoses were made using the DSMIII-R criteria by a senior neurologist, and the diagnosis of a major depression by a psychiatrist according to DSM-IV [20], [21]. The list of chronic diseases included in the calculation of multimorbidity is shown in (Table S1).

Of the 390 study participants included in these analyses, 86 persons (20 of 149 without disease and 66 of 269 with one disease at baseline) died before the follow-up assessment and for these individuals the presence of multimorbidity was calculated on the basis of the inpatient registry data only.

### Incident multimorbidity

Multimorbidity was the primary outcome measure in this study and was defined as any co-occurrence of two or more chronic conditions in the same individual, whether coincidental or not [10]. Incident cases were defined as subjects with no or only one chronic disorder at baseline who developed at least another chronic disease during the 3-year follow-up.

### Possible predictors of multimorbidity

We examined the following variables:

- Social demographic measures: age (years), sex, living situation, living arrangement, and education. Education was measured by the maximum years of formal schooling, and this variable was dichotomized (≥8 versus <8 years) according to our previous study [2]. Living arrangement was dichotomized into living alone or living with others.

Lifestyle related factors: physical activity, smoking, and alcohol drinking. Physical activity was assessed approximately three years before the baseline of the current study, and was dichotomized in the analyses into ‘no physical activity’ and ‘at least weekly physical activity’. Smoking was operationalized in the analyses in three classes. Alcohol drinking was operationalized in the analyses as ‘less than 1 unit per week’, ‘between 1 and 7 units per week’, and ‘7 or more units per week’.

- Physical examination variables: Body Mass Index (BMI, kg/m^2^), heart rate (beats/min), blood pressure (mmHg), and the six Katz items on Activities of Daily Living [15];
- Laboratory findings: hemoglobin (Hb, g/l), albumin (Alb, g/l), erythrocyte sedimentation rate (ESR, mm/hour), and white blood cell count (WBC, *10^9^ leukocytes/l);
- Affective and cognitive scales: the Comprehensive Psychopathological Rating Severity scale for mood related and motivation related symptoms of depression (CPRS, range 0-48, where higher score indicates more symptoms; [16]); global cognition using the Mini Mental State Examination (MMSE, range 0-30, where higher score indicate better cognition; [17]). CPRS is a structured psychiatric interview with both directed questions and observations. The CPRS severity subscale for mood-related symptoms assesses the following four symptoms on a scale from 0-6: dysphoria, appetite disturbance, feelings of guilt, and thoughts of death. The severity subscale for motivation-related symptoms assesses: lack of interest, psychomotor change, loss of energy, and concentration difficulties, again scoring 0-6 per item. If the participant was not able to answer the questions reliably, an informant was contacted.

### Statistical analysis

Using descriptive statistics the baseline characteristics were presented grouping the participants according to the presence of no or one chronic disease. Cumulative incidence for multimorbidity was calculated by the number of new cases of multimorbidity during the follow-up period divided by the number of subjects at risk in the population at the beginning of the study and not lost to follow up due to withdrawal of informed consent; 95% confidence intervals were calculated according to the binomial distribution. Multimorbidity incidence rates were calculated as the number of new cases of multimorbidity divided by the total person-time observed between the two assessments or until date of death. Exact Poisson rate 95% confidence intervals were calculated. Logistic regression analysis was used to study the effect of the possible predictors of multimorbidity, starting with univariate analyses checking the effect of each variable separately, followed by bivariate analyses taking into account the effect of the presence of one disease at baseline and next by adding an interaction term between the predictor of interest and the presence of one disease. Since the interaction analyses showed that the presence of disease at baseline was likely an effect modifier, the multivariate analyses were stratified for the presence or absence of disease at baseline. All missing values in the variables that were used in the analyses for this study – potential predictors as well as the dependent variables – were imputed twenty times assuming a multivariate normal distribution of the data using SAS Proc MI and SAS Proc MIANALYZE to calculate measures of precision. All results reported in this paper are based on the imputed datasets. A series of sensitivity analyses were run.

## Results

**Figure 1:**
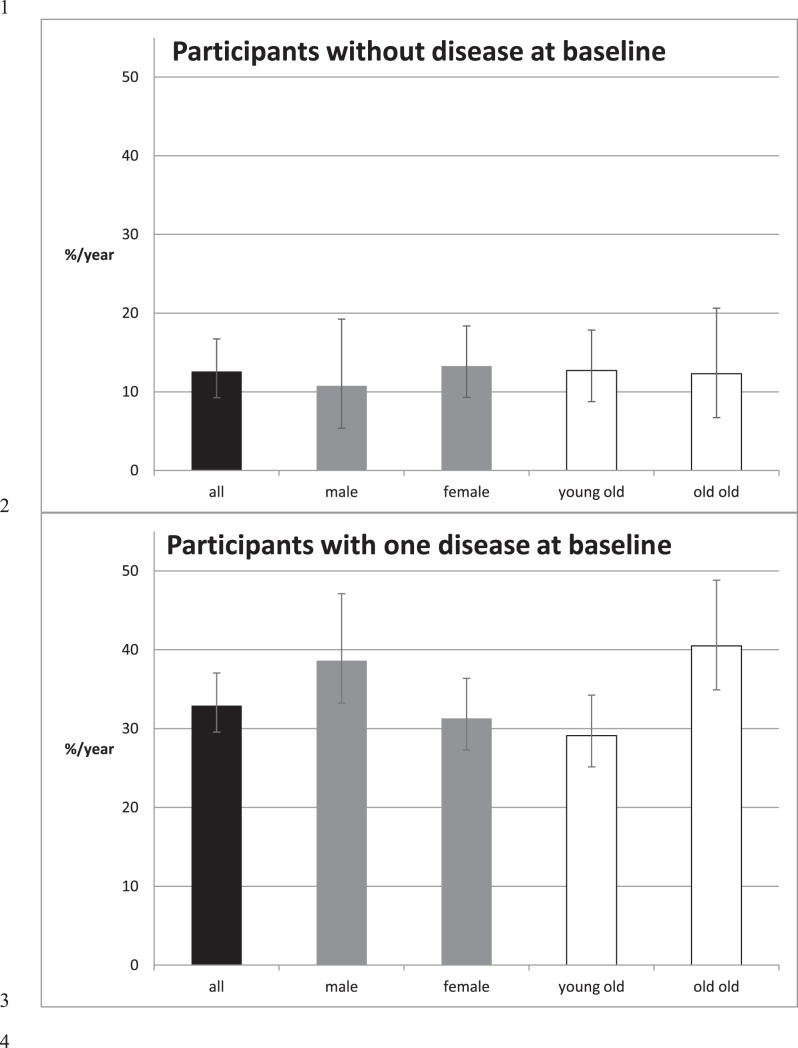
Multimorbidity (two or more diseases) incidence rates (95% confidence intervals) for participants without and with one disease at baseline (black) and by sex (grey) and age category (young old: <85 years and old old: ≥85 years, white).

At baseline 418 participants were living independently and were not affected by multimorbidity. As 28 subjects refused the follow-up assessment, 390 participants were included in the analysis: of these, 140 participants had no disease and 250 participants had one disease at the baseline (Table 1). After three years, among the 140 participants who were without any chronic disease at baseline 50 (35.7%; 95% CI = 27.8–43.7) still were without disease, 43 (30.7%; 95% CI = 23.1–38.4) had one disease and 47 (33.6%; 95% CI = 25.8–41.4) developed multimorbidity. The 3-year cumulative incidence for multimorbidity in this group was 33.6%. In the subgroup of the 250 participants with one disease at baseline, 22 (8.8%; 95% CI = 5.3–12.3) were without morbidity, 62 (24.8%; 95% CI = 19.5–30.2) still have one disease and 166 (66.4%; 95% CI = 60.5–72.3) developed multimorbidity. The 3-year cumulative incidence for multimorbidity in this group was 66.4%. The incidence rate was 12.6 per 100 person-years (95% CI: 9.2–16.7) and 32.9 per 100 person-years (95% CI: 28.1–38.3), respectively. Further, we calculated the incidence rates separately for the two subgroups (subjects with one or no chronic disease at baseline), and stratified by gender and age. Results are reported in Figure 1. The risk of developing multimorbidity was higher among subjects with one disease and older people; no substantial gender differences were detected.

**Table 1:** Baseline characteristics of the participants according to the presence/absence of one chronic disease.

	No chronic disease n = 140	One chronic disease N = 250
Age (year), mean (sd)	83.3 (3.7)	84.2 (4.2)
Female, n (%)	102 (73)	190 (76)
Low education (<8 years), n (%)	58 (42)	110 (44)
MMSE, mean (sd)	27 (2)	26 (4)
Living alone*, n (%)	102 (73)	179 (72)
At least one ADL disability, n (%)	21 (15)	58 (23)
Physically active, n (%)	30 (21)	28 (11)
Smoking, n (%)		
Non-smoker	83 (61)	141 (62)
Former smoker	24 (18)	59 (26)
Current smoker	29 (21)	27 (12)
Alcohol drinking, n (%)		
<1 unit/week	88 (63)	165 (69)
1–6 units/week	48 (35)	66 (28)
≥7 units/week	4 (3)	7 (3)
Body Mass Index, n (%)		
<18.5 kg/m^2^	6 (5)	16 (8)
18.5–25 kg/m^2^	93 (75)	127 (64)
>25 kg/m^2^	25 (20)	57 (29)
Heart rate, n (%)		
≤60 bpm	20 (14)	26 (11)
61–100 bpm	115 (83)	214 (87)
≥100 bpm	3 (2)	6 (2)
Diastolic blood pressure (mmHg), mean (sd)	80 (11)	81 (11)
Systolic blood pressure (mmHg), mean (sd)	158 (20)	160 (21)
CPRS, mean (sd)	2.1 (2.4)	2.1 (2.8)
Hemoglobin (g/l), mean (sd)	138 (9)	137 (13)
WBC (*10^9^ leucocytes/l), mean (sd)	6.5 (1.5)	6.9 (2.1)
ESR (mm/hour), mean (sd)	16 (14)	19 (15)
Albumin (g/l), mean (sd)	42.2 (2.3)	42.3 (2.8)

*As opposed to living independently but with others.

ADL, Activities of Daily Living (measured with Katz ADL, 0–6, higher score indicates worse function).

CPRS, Comprehensive Psychopathological Rating Severity (0–48, higher score indicates more depressive symptoms).

ESR, Erythrocyte Sedimentation Rate.

MMSE, Mini-Mental State Examination (0–30, higher score indicates better function).

WBC, white blood cell.

In the univariate models several factors were associated with an increased relative odd of incident multimorbidity at follow up in the whole group of 390 participants (Table 2). As already said in the methods, the interaction between potential predictors of incident multimorbidity and the presence of one disease at baseline was statistically significant for almost all variables. Thus, all analyses were run after stratification for absence or presence of one disease at baseline.

**Table 2:** The crude odds ratios (OR) and 95% confidence intervals (95% CI) of potential predictors for multimorbidity separately for the two subgroups of people with no or one disease at baseline.

Predictor of interest	No chronic disease at baseline (n = 140)	One chronic disease at baseline (n = 250)
	OR (95% CI)*	OR (95% CI)*
Age (years, continuous)	1.00 (0.91–1.10)	1.08 (1.01–1.16)**
Sex (female *vs* male)	1.34 (0.60–3.01)	0.73 (0.38–1.37)
Education (low *vs* high education)	1.81 (0.89–3.69)	1.22 (0.72–2.08)
Living alone *vs* living with other	1.34 (0.60–3.01)	1.06 (0.59–1.90)
Katz ADL (number of disabilities, continuous)	0.76 (0.27–2.11)	1.29 (0.85–1.94)
Hemoglobin (g/l, continuous)	0.97 (0.93–1.01)	1.00 (0.98–1.02)
ESR (mm/hour, continuous)	1.05 (1.01–1.08)**	1.01 (0.99–1.03)
WBC* (10^9^ leucocytes/l, continuous)	1.18 (0.92–1.51)	1.08 (0.94–1.25)
Albumin (g/l, continuous)	1.05 (0.90–1.21)	0.97 (0.88–1.07)
Heart rate		
≤60 *vs* 60–99 bpm	1.81 (0.69–4.75)	0.97 (0.41–2.28)
≥100 *vs* 60–99 bpm	4.18 (0.37–47.66)	1.07 (0.19–5.98)
Diastolic blood pressure (increase of 10 mmHg)	0.71 (0.49–1.03)***	0.91 (0.72–1.16)
Systolic blood pressure (increase of 10 mmHg)	0.92 (0.77–1.11)	0.99 (0.88–1.12)
Physical activity (yes *vs* no)	0.42 (0.16–1.11)***	1.30 (0.55–3.09)
Depressive symptoms (CPRS, continuous)	1.16 (1.00–1.35)**	1.04 (0.94–1.15)
Worse cognitive abilities (MMSE, continuous)	1.25 (1.06–1.48)**	1.06 (0.99–1.14)***
Smoking (3 categories: never, former, current, continuous)	0.92 (0.59–1.43)	1.14 (0.77–1.70)
Alcohol drinking		
<1 unit/week *vs* 1–6 units/week	1.02 (0.48–2.17)	1.89 (1.05–3.40)**
≥7 units/week *vs* 1–6 units/week	19.44 (0.70–538.9)***	3.85 (0.52–28.4)
BMI		
<18.5 *vs* 18.5–25 kg/m^2^	1.07 (0.20–5. 78)	0.93 (0.31–2.76)
>25 *vs* 18.5–25 kg/m^2^	1.26 (0.49–3.25)	1.26 (0.69–2.38)

*the odd ratios presented are for the continuous and ordinal variables the odds ratio for an increase of one unit/level unless stated otherwise and for the dichotomized variables for presented dichotomy.

**p<0.05.

***p<0.10.

ADL, activities of daily living (0–6, higher score indicates worse function).

CPRS, Comprehensive Psychopathological Rating Severity (0–48, higher score indicates more depressive symptoms).

BMI, Body Mass Index.

ESR, erythrocyte sedimentation rate.

MMSE, mini-mental state examination (0–30, higher score indicates better function).

WBC, white blood count.

The multivariate models combined potential predictors, and confounders as they were derived from the univariate analyses and the analyses were stratified for the presence or absence of one disease at baseline (Table 3). In the final multivariate adjusted model for persons without any disease at baseline, global cognition (MMSE) was significantly (OR 1.22, 95% confidence interval 1.00–1.48, p = 0.05) related to multimorbidity occurrence and mood (CPRS) almost (OR 1.18, 95%-CI 0.99–1.40, p = 0.06). In the multivariate adjusted model for persons with already one disease at baseline, only higher age was significantly related to multimorbidity incidence at follow up (table 3; OR 1.09, 1.01–1.17, p = 0.03). No further statistically significant predictors of multimorbidity incidence were identified in this stratum, however, drinking less than 1 unit of alcohol per week was associated with an OR of 1.87 (0.98–3.58) at p = 0.06.

**Table 3:** Association between potential predictors and the incidence of multimorbidity in persons with and without one chronic disease at baseline.

Predictors of interest	No chronic disease at baseline (n = 140)	One chronic disease at baseline (n = 250)
	OR (95% CI)*	OR (95% CI)*
Age (years, continuous)	0.99 (0.89–1.11)	1.09 (1.01–1.17)**
Sex (female *vs* male)	0.87 (0.30–2.52)	0.88 (0.42–1.87)
Education (low *vs* high education)	1.36 (0.56–3.32)	0.94 (0.51–1.75)
Katz ADL (number of disabilities, continuous)	0.80 (0.25–2.60)	1.01 (0.64–1.60)
Hemoglobin (g/l, continuous)	0.98 (0.93–1.03)	1.01 (0.99–1.04)
ESR (mm/hour, continuous)	1.02 (0.99–1.06)	1.02 (0.99–1.04)
WBC (*10^9^ leucocytes/l, continuous)	1.19 (0.89–1.58)	1.06 (0.92–1.22)
Diastolic blood pressure (increase of 10 mmHg)	0.98 (0.94–1.01)	0.99 (0.96–1.02)
Physical activity (yes *vs* no)	0.44 (0.15–1.28)	1.55 (0.61–3.92)
Depressive symptoms (CPRS, continuous)	1.18 (0.99–1.40)***	1.03 (0.93–1.14)
Worse cognitive abilities (MMSE, continuous)	1.22 (1.00–1.48)**	1.04 (0.96–1.12)
Smoking (3 categories: never, former, current, continuous)	0.86 (0.49–1.52)	1.29 (0.80–2.07)
Alcohol drinking		
<1 unit/week *vs* 1–6 units/week	0.90 (0.36–2.22)	1.87 (0.98–3.58)***
≥7 units/week *vs* 1–6 units/week	11.43 (0.37–348.43)	2.89 (0.36–22.97)
BMI		
<18.5 kg/m^2^ *vs* 18.5–25 kg/m^2^	1.30 (0.11–7.98)	0.95 (0.30–3.03)
>25 kg/m^2^ *vs* 18.5–25 kg/m^2^	1.48(0.47–4.71)	1.33 (0.68–2.60)

Odds Ratio (OR) and 95% confidence intervals (95% CI) from multivariate logistic regression models.

*the odd ratios presented are for the continuous and ordinal variables the odds ratio for an increase of one unit/level unless stated otherwise and for the dichotomized variables for presented dichotomy.

**p<0.05.

***p<0.10.

ADL, activities of daily living (0–6, higher score indicates worse function).

BMI, Body Mass Index.

CPRS, Comprehensive Psychopathological Rating Severity (0–48, higher score indicates more depressive symptoms).

ESR, erythrocyte sedimentation rate.

MMSE, mini-mental state examination (0–30, higher score indicates better function).

WBC, white blood count.

### Sensitivity analysis

As global cognition (MMSE) and mood (CPRS) turned out to be likely predictors of multimorbidity, we reran the analyses with dementia and depression not counted for the incidence of multimorbidity at follow up in order to rule out the effect of these two predictors. The results showed that the correlations of MMSE and CPRS score with multimorbidity were comparable. The association with MMSE diminished slightly to OR 1.15 (0.93–1.41, p = 0.19). The association with CPRS increased slightly: OR 1.20 (1.00–1.44, p = 0.05). Further, we calculated the associations of MMSE quartile scores as categorical variable with multimoribidity occurrence (best performing quartile as the reference group) for the two subgroups with and without morbidity at baseline. These results suggest that it is perhaps the two lowest quartiles (an MMSE below 27) that have the strongest association with multimorbidity occurrence in the group without disease at baseline (Table S2).

## Discussion

A systematic review of the literature on multimorbidity [1] identified very few prospective studies which evaluated incidence and predictors of multimorbidity. In the present study, we used data on the older population enrolled in the Kungsholmen Project to quantify the incidence of multimorbidity over a period of three years. In this 78+ year-old population, multimorbidity had a high incidence as it developed with a rate of approximately 12 per 100 person-years in people not affected by any chronic disorders and 33 per 100 person-years in those with already one disease at baseline. Secondly, among the few factors identified as possible predictors of multimorbidity onset, clinical symptoms related to mental disorders emerged as the most important factors to identify people at risk to develop multimorbidity among subjects without any disease. Lower cognitive abilities and perhaps also increasing number of depression-related symptoms predicted the development of multimorbidity in persons with no disease at baseline. Among participants with already one disease, only age was a predictor of multimorbidity incidence.

In 1998, van den Akker and colleagues reported that cumulative incidence of multimorbidity, defined as 2 or more new diagnoses during one year, was 1.3% in the general population (all ages) but >5% in those aged 80 and older [10]. However, the authors did not select persons according to the presence of none or one plus disease at baseline. In our study, the cumulative incidence of multimorbidity in very old elderly differed depending on the absence or presence of one disease at baseline. In fact, two third of participants affected by already one disease became multimorbid after only 3 years compared to one third of those without any disease. Anyhow even taking into account this last group, the estimates of annual cumulative incidence and the incidence rates were double higher than those reported by van den Akker [10]. Dissimilarities in the diagnoses recording (van den Akker evaluated multimorbidity in a general practice setting) may explain this difference.

Despite the fact than more than 50% of 75+ old subjects are affected by multimorbidity, little research has focused on risk factors and predictors of this syndrome. A couple of papers focused on the impact of socioeconomic status on the development of multimorbidity [11], [22]. Childhood financial hardship and lifetime earnings were associated with multimorbidity being the latter a protective factor [22] and lower education was associated with multimorbidity [11]. In this study using data from the Kungsholmen population that is over age 75 we found that education, which is a good indicator of SES, education was positively associated with incident multimorbidity, although without reaching statistical significance, especially in participants with no disease at baseline. In the Kungsholmen Project, data on another indicator of SES were collected, e.g. lifetime occupation. Due to the high correlation between occupation-based SES and education in this population we did not use the occupation variable in the present study. In fact, previous analyses on prevalent multimorbidity in the Kungsholmen Project showed that occupation-based SES had a crude association with multimorbidity, but not when adjusted for education. Besides, low education showed a strong association with multimorbidity independent of high or low occupation-based SES [2].

We found that at higher age cognition and depressive symptoms were possible predictors of multimorbidity onset in people who were without significant chronic morbidity at baseline. In the older population, depressive symptoms are commonly associated with chronic medical conditions. The finding was confirmed – even strengthened slightly – when we excluded depression diagnosis from the multimorbidity definition. Depression can delay diagnosis of other diseases and negatively affect medication adherence and healthy behaviors to prevent other clinical conditions [23]. Moreover, depressive symptoms may lead to social isolation. Van den Akker and colleagues found that living in a family compared to living alone and a large social network were protective factors for multimorbidity occurrence [13]. This result is consistent with previous studies showing that older age depression was associated with several adverse outcomes if not adequately treated, such as increased risk of disability, poor quality of life and mortality [24], [25]. On the other hand, chronic diseases increase the risk of depression, with the prevalence of depression being up to five times higher in persons with chronic medical conditions [26]. This strong association can be explained by the presence of disability, pain and polypharmacy in the elderly affected by multiple diseases. In this study, depressive symptoms were measured three years before the diagnosis of multimorbidity, thus the possibility of such reverse causation seems unlikely. Finally, the mechanism linking depressive symptoms to incident multimorbidity may be chronic inflammation. In fact, several studies have shown higher circulating pro-inflammatory cytokines levels in depressive disorders [27]. Unfortunately, in the Kungsholmen Project such markers of inflammation were not available.

Further, a worse MMSE was a predictor of the occurrence of multimorbidity in those participants free from any disease. So far, cognitive impairment has mainly been studied as a consequence of several chronic diseases and also of multimorbidity [28]. Previous cross-sectional and longitudinal studies reported a positive association between poor health and multimorbidity and cognitive complaints or impairment [29], [30]. In our study, worse cognition measured three years before the multimorbidity assessment was associated with the development of multimorbidity. It is possible that cognitive decline is a manifestation of a latent and still undiagnosed chronic disease such as low oxygen levels in chronic respiratory diseases, insulin imbalance in diabetes, and thyroid hormone deficiency or excess in thyroid dysfunction may have already affected the brain [31]. Silent brain infarction may also be present. Another explanation is that cytokine activated immune system dysregulation as well as chronic stress, both frequent in the elderly, may affect first the brain and secondly the body through the imbalance of the adrenocortico axis [32]. Finally, the same disease pathology or risk factors may lead to both cognitive impairment and specific chronic disease; for example estrogen deficiency may be associated with both cognitive impairment and osteoporosis.

Among characteristics that we did not find associated with incident multimorbidity, there were some life-style factors usually correlated with chronic diseases, such as smoking and alcohol. Indeed, the leading chronic-disease causes of death can be attributed to tobacco use and alcohol consumption beyond physical inactivity [33]. Our findings may be due to a survival effect. In fact, subjects who are continuous smokers have a high mortality risk and many of them probably died before the enrolment in the present study. The lack of the expected association of alcohol intake with multimorbidity can be also being due to the common underrating of alcohol intake in interviews. Remarkably, in people who had one disease at baseline already, drinking less than one unit of alcohol per week was associated with an increased occurrence of multimorbidity three years later as compared to moderate drinkers. We want to be cautious interpreting this finding, however, it could be that non-drinkers actually stopped drinking due to their health condition. Thus, they might be at higher risk for increasing disease burden than moderate drinkers. It has been suggested that moderate “alcohol consumption may represent a marker of higher social level [and] superior health status” [34].

### Limitations

Because of the study design, characteristics of the patients were assessed only in a single measurement at baseline. Some factors, such as physical activity, are considered relatively stable, but they can change over time, especially as a result of a disease. In line with previous studies [12], [35], a follow-up time of three years was chosen for the calculation of incident cases. In fact, distribution of diseases is not random in time, but disease susceptibility is markedly different according to the duration of the follow-up interval, and the stability of several individual characteristics over time is not known [12]. Second, we pre-selected a list of 39 chronic diseases, thus automatically excluding some others (although rare); moreover, for a number of participants we had to rely only on hospital registry information, which may differ in some way from those who were seen by the study physician for a second time, although this is also a strength for those who died in between intervals and could still be included thanks to the registry information.

### Conclusions

Multimorbidity has a high incidence in the elderly, especially in those with already one disease at baseline. Worse cognition and low mood emerged as possible predictors of development of multimorbidity, suggesting a strong influence of mental disorders on the health of older people. Due to increasing life expectancy, prevalence of chronic diseases is expected to increase in the near future [36]. The coexistence of multiple chronic diseases has been found to be associated with several adverse outcomes as well as high medical care costs. Identifying at risk subjects to develop multimorbidity may help to implement prevention strategies among older people.

## Supporting Information

Table S1 **List of chronic diseases included in the calculation of multimorbidity (chronic diseases present at least once in the complete KP cohort of participants who participated in the first follow up).**
(DOCX)

Table S2 **The crude odds ratios (OR) and 95% confidence intervals (95% CI) of MMSE quartile scores for multimorbidity separately for the two subgroups of people with no or one disease at baseline with the people in Q1 (MMSE 29–30) as reference category (on unimputed dataset, because there were no missings for the variables involved).**
(DOCX)
