# Supplementary material for: Incidence and Predictors of Multimorbidity in the Elderly: A Population-Based Longitudinal Study
Source: PLoS One. 2014 Jul 24;9(7):e103120. doi: 10.1371/journal.pone.0103120 (PMC4109993; doi:10.1371/journal.pone.0103120)
Supplement: Table S1 — List of chronic diseases included in the calculation of multimorbidity (chronic diseases present at least once in the complete KP cohort of participants who participated in the first follow up). (DOCX) [file pone.0103120.s001.docx]

**Table S1.** List of chronic diseases included in the calculation of multimorbidity (chronic diseases present at least once in the complete KP cohort of participants who participated in the first follow up).

| **Diseases** |
| --- |
| Alcohol dependence syndrome |
| Anemia |
| Rheumatoid arthritis |
| Cancer |
| Cardiomiopathy |
| Cerebrovascular diseases |
| cholelithiasis |
| Calculus of kidney/ureter |
| Colitis |
| Chronic obstructive pulmonary disease |
| Chronic pulmonary heart disease |
| Chronic renal failure |
| Chronic rheumatic heart disease |
| Crystal arthropathies |
| Deafness |
| Depression |
| Diabetes |
| Diverticula of intestine |
| Liver diseases |
| Pancreas diseases |
| Atrial fibrillation |
| Epilepsy |
| Disorders of the eyes |
| Functional digestive disorders |
| Heart failure |
| Hip fracture |
| Hyperplasia of prostate |
| Hypertension |
| Ischemic heart disease |
| Intestinal malabsorption |
| Lung diseases due to external agents |
| Migraine |
| Arthritis |
| Parkinson disease |
| Peripheral nerve system diseases |
| Schizophrenia |
| Thyroid disorders |
| Dementia |
| Rheumatic Polymyalgia |
