# Supplementary material for: Incidence and Predictors of Multimorbidity in the Elderly: A Population-Based Longitudinal Study
Source: PLoS One. 2014 Jul 24;9(7):e103120. doi: 10.1371/journal.pone.0103120 (PMC4109993; doi:10.1371/journal.pone.0103120)
Supplement: Table S2 — The crude odds ratios (OR) and 95% confidence intervals (95% CI) of MMSE quartile scores for multimorbidity separately for the two subgroups of people with no or one disease at baseline with the people in Q1 (MMSE 29–30) as reference category (on unimputed dataset, because there were no missings for the variables involved). (DOCX) [file pone.0103120.s002.docx]

**Table S2** The crude odds ratios (OR) and 95% confidence intervals (95% CI) of MMSE quartile scores for multimorbidity separately for the two subgroups of people with no or one disease at baseline with the people in Q1 (MMSE 29-30) as reference category (on unimputed dataset, because there were no missings for the variables involved).

| Predictor of interest | No chronic disease at baseline  (n=140) | One chronic disease at baseline (n=250) |
| --- | --- | --- |
|  | OR (95% CI) | OR (95% CI) |
| MMSE quartile score*  Q2, MMSE 27-28 | 1.04 (0.42 – 2.61) | 0.68 (0.34-1.37) |
| Q3, MMSE 25-26 | 2.70 (0.98 – 7.49) | 0.87 (0.38-1.96) |
| Q4, MMSE LE 24 | 3.27 (1.01 – 10.58) | 1.39 (0.63-3.09) |

*Q1 (MMSE of 29-30) is the reference group

MMSE, mini-mental state examination (0-30, higher score indicates better function)
